# Supplementary material for: Adsorption Behavior and Relative Distribution of Cd2+ Adsorption Mechanisms by the Magnetic and Nonmagnetic Biochars Derived from Chicken Manure
Source: Int J Environ Res Public Health. 2020 Mar 2;17(5):1602. doi: 10.3390/ijerph17051602 (PMC7084209; doi:10.3390/ijerph17051602)
Supplement: Supplementary file 1 [file ijerph-17-01602-s001.pdf]

Supplemental materials

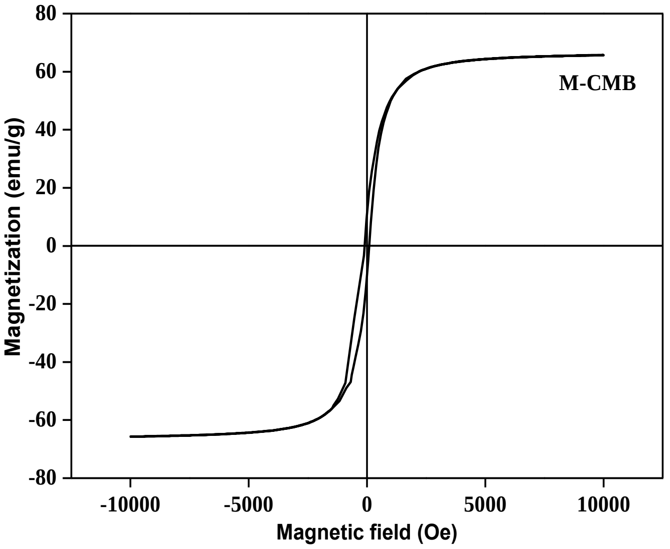

Fig. S1. Magnetization curves of M-CMB at room temperature.

Table S1. Kinetic parameters of adsorption for Cd^{2+} by both biochars at different initial metal concentrations.

C_0 (mg/L)	Biochar	Pseudo-second-order kinetic				Pseudo-first-order kinetic		
		K_2 (g/mg/min)	R^2	$Q_{e,cal}$ (mg/g)	$Q_{e,exp}$ (mg/g)	K_1 (min^{-1})	R^2	$Q_{e,cal}$ (mg/g)
20	CMB	0.01	0.9550	16.43	16.07	0.06	0.8344	15.23
	M-CMB	0.01	0.9545	9.16	8.71	0.04	0.8684	7.32
50	CMB	0.13	0.9782	29.62	28.27	0.08	0.9047	26.85
	M-CMB	0.01	0.9696	20.39	20.18	0.09	0.8387	19.17
100	CMB	0.05	0.9924	40.07	39.40	0.04	0.9168	35.85
	M-CMB	0.01	0.9742	27.58	26.45	0.03	0.9029	23.03

C_0 is the initial concentration of Cd^{2+}, mg/L. $Q_{e,cal}$ is the adsorption capacity at equilibrium calculated by kinetic model, mg/g. $Q_{e,exp}$ is the adsorption capacity at the experimental conditions, mg/g.

Table S2. Isotherm parameters of adsorption for Cd^{2+} by both biochars under different temperatures.

Temperature (K)	Biochar	Freundlich isotherm			Langmuir isotherm		
		K_F (L/g)	1/n	R^2	$Q_{m,cal}$ (mg/g)	K_L (L/mg)	R^2
293	CMB	18.65	0.18	0.9869	40.73	0.59	0.7457
	M-CMB	3.26	0.45	0.9515	37.21	0.03	0.9071
303	CMB	20.88	0.18	0.9940	42.98	1.39	0.7826
	M-CMB	15.48	0.13	0.9967	26.44	1.90	0.6740
313	CMB	25.84	0.19	0.9974	54.72	1.14	0.8384
	M-CMB	11.94	0.26	0.9500	39.89	0.19	0.8946

$Q_{m,cal}$ is the maximum adsorption capacity calculated by Langmuir isotherm model, mg/g.
